# Brain-Resident Microglia and Blood-Borne Macrophages Orchestrate Central Nervous System Inflammation in Neurodegenerative Disorders and Brain Cancer

**DOI:** 10.3389/fimmu.2018.00697

**Published:** 2018-04-06

**Authors:** Lisa Sevenich

**Affiliations:** Georg-Speyer-Haus, Institute for Tumor Biology and Experimental Therapy, Frankfurt am Main, Germany

**Keywords:** neuroinflammation, tissue-resident macrophages, microglia, neurodegeneration, cancer

## Abstract

Inflammation is a hallmark of different central nervous system (CNS) pathologies. It has been linked to neurodegenerative disorders as well as primary and metastatic brain tumors. Microglia, the brain-resident immune cells, are emerging as a central player in regulating key pathways in CNS inflammation. Recent insights into neuroinflammation indicate that blood-borne immune cells represent an additional critical cellular component in mediating CNS inflammation. The lack of experimental systems that allow for discrimination between brain-resident and recruited myeloid cells has previously halted functional analysis of microglia and their blood-borne counterparts in brain malignancies. However, recent conceptual and technological advances, such as the generation of lineage tracing models and the identification of cell type-specific markers provide unprecedented opportunities to study the cellular functions of microglia and macrophages by functional interference. The use of different “omic” strategies as well as imaging techniques has significantly increased our knowledge of disease-associated gene signatures and effector functions under pathological conditions. In this review, recent developments in evaluating functions of brain-resident and recruited myeloid cells in neurodegenerative disorders and brain cancers will be discussed and unique or shared cellular traits of microglia and macrophages in different CNS disorders will be highlighted. Insight from these studies will shape our understanding of disease- and cell-type-specific effector functions of microglia or macrophages and will open new avenues for therapeutic intervention that target aberrant functions of myeloid cells in CNS pathologies.

## Introduction

The brain has long been regarded as an immunologically privileged site in which the presence of the blood–brain barrier (BBB) restricts the entry of blood-borne immune and inflammatory cells to the central nervous system (CNS) [for review, see Ref. (1)]. Consequently, key functions in tissue homeostasis and immune defense were attributed to brain-resident cell types, such as microglia or astrocytes (2, 3). Microglia are regarded as the innate immune cell of the CNS. As part of their routine surveillance, microglia continuously monitor their surrounding with motile protrusions to sense and resolve any disturbance (4). Along with their well-established role as immediate responders to injury and infection (5, 6), there has been an increasing appreciation of the importance of microglia for normal CNS development and function, including developmentally regulated neuronal apoptosis, neurogenesis, myelogenesis, and synaptic pruning (7–9). Given their central role in CNS inflammation, it is not surprising that dysregulation of microglial activation and microglia-induced inflammation is observed in virtually all brain malignancies, including neurodegenerative disorders as well as primary and metastatic brain cancers. Blood-borne immune and inflammatory cells have recently emerged as an important component of the disease-associated microenvironment in the brain and are regarded as critical mediators of progression in neurodegenerative disease and brain cancers. However, the lack of experimental systems that distinguish between recruited and brain-resident myeloid cells has previously halted analysis of cell-type-specific functions in CNS inflammation. The development of new methodologies provides unprecedented opportunities for comprehensive in-depth analyses of the immune landscape of the CNS under steady-state and pathological conditions. Single-cell RNAseq or mass cytometry (CyTOF) allow for an unbiased view on the immune milieu of the brain parenchyma and adjacent boundaries. In addition to the well-characterized macrophage populations of non-parenchymal areas of the brain (10), it is increasingly recognized that various immune cell populations including a large diversity of lymphoid and myeloid subpopulations are present in particular in the meninges and the choroid plexus (11–14). Analysis of parenchymal myeloid cells also revealed high cellular heterogeneity. The existence of distinct myeloid cell phenotypes may reflect functional diversity, different ontological origins, or various cell differentiation states already at steady state (11). The question how environmental cues in different brain malignancies sculpt transcriptional profiles and epigenetic states of microglia and recruited myeloid cell populations during disease progression has recently gained attention. A growing number of studies seek to unravel the heterogeneity of the disease-associated immune landscape to functionally link different cell states to disease progression. Detailed knowledge of the impact of individual cell populations or activation states across different CNS malignancies is critical for the development of improved therapeutic strategies to target dysfunctional cells without affecting essential physiological or beneficial functions. The aim of this review is to discuss recent insights into the cellular and molecular identity of the heterogeneous population of cerebral myeloid cells in different CNS disorders to highlight common and unique features of the distinct subpopulations in the respective CNS pathologies.

## Ontological Origin of Myeloid Cells in the CNS in Health and Disease

Microglia, the brain-resident macrophages, represent the largest population of myeloid cells in the CNS and are localized in the brain parenchyma. The term microglia was first coined by Pio del Rio-Hortega to describe the non-neural, non-astrocytic “third element of the nervous system” that is distinct from neuroectodermal oligodendroglia and oligodendrocytes. Del Rio-Hortega’s findings indicated a mesodermal origin of microglia [for historical review, see Ref. (15)]. However, there was a long-lasting debate on the ontological origin of microglia. An alternative hypothesis proposed that microglia originate from neuro-ectodermal-derived glioblasts (16). This theory was seemingly supported by the findings that donor bone marrow cells failed to contribute to the adult microglia population in either newborn (17) or adult rodents (18). Hickey and Kimura demonstrated that in bone marrow chimera only perivascular microglia derived from the bone marrow (19). The authors used the term perivascular microglia for the cell population that to date is referred to as perivascular macrophages that are located in the Robin-Virchow space. Further evidence that resident microglia are not replaced by cells from the bone marrow was provided by Lassmann et al. (20). The definitive proof for a mesodermal origin of microglia was achieved through a genetic study that showed that mice lacking the crucial transcription factor for myeloid cells, PU.1, are devoid of microglia (21, 22).

**Figure 1:**
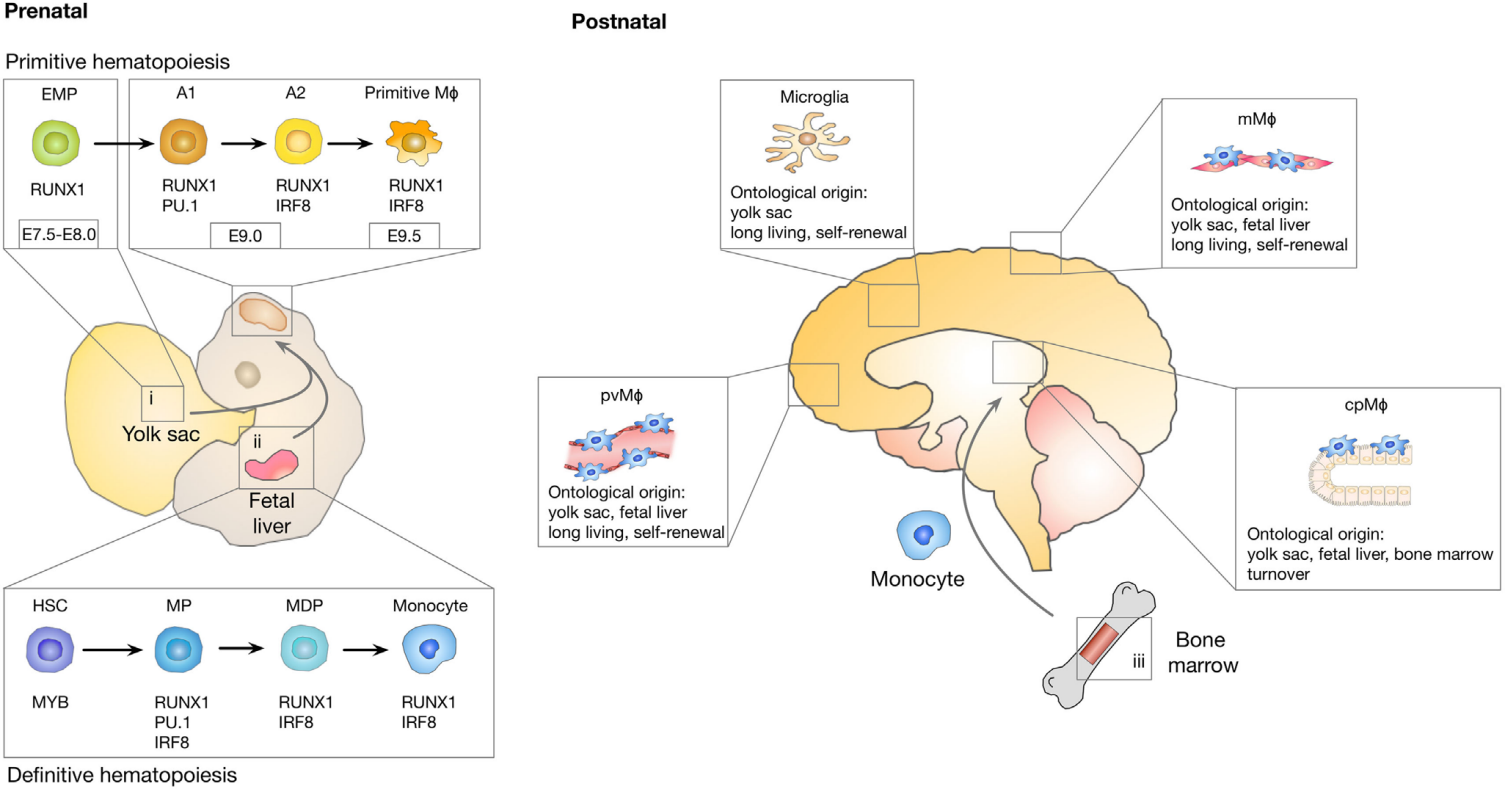
Ontological origin of macrophage subpopulations in the central nervous system (CNS). A first wave of myeloid cell development takes place in the yolk sac (i) between E7.0 and E8.0 in a process known as primitive hematopoiesis that leads to the generation of erythro-myeloid progenitor (EMP) cells. EMP cells give rise to A1 (cKit^+^Cx3cr1^−^) cells followed by A2 (Cx3cr1^+^) cells that differentiate into microglia, perivascular macrophages (pvMϕ), meningeal macrophages (mMϕ), and choroid plexus macrophages (cpMϕ). Microglia originate exclusively from yolk sac-derived progenitors, while non-parenchymal CNS macrophages are replenished with fetal liver-derived progenitor cells (ii) as part of definitive hematopoiesis. Perinatally, hematopoiesis starts to be restricted to the bone marrow (iii). Among the CNS macrophages, cpMϕ are the only population with substantial constitution from bone marrow progenitors. Microglia, pvMϕ, and mMϕ are considered to be long-living cells that regenerate through self-renewal.

Even after the myeloid origin of microglia was proven, debate about the nature of microglia progenitors remained. Controversy was mainly caused by the fact that there are two major sites of hematopoiesis during embryogenesis: the yolk sac and the fetal liver. As depicted in Figure 1, primitive hematopoiesis in mice is initiated in the yolk sac at around E7.0, which leads predominantly to the generation of macrophages and erythrocytes (23). Yolk sac-derived primitive macrophages enter the embryo proper after the circulatory system has been established (from E8.5 to E10) (24) and populate various organs that contain tissue-resident macrophage populations, including the brain. Population of the fetal brain by primitive macrophages takes place before the onset of monocyte production by the fetal liver and before the establishment of the BBB. A second wave of “definitive” hematopoiesis is initiated by hematopoietic progenitors that are generated in the yolk sac and the AGM (aorta, gonads, and mesonephros) region of the embryo proper and that migrate into the fetal liver around E10.5. After E11.5, the fetal liver serves as the major hematopoietic organ and generates all hematopoietic linages including monocytes (25). In contrast to primitive hematopoiesis, definitive hematopoiesis depends on the transcription factor Myb (26). Around birth, hematopoiesis starts to be restricted to the bone marrow (27). It further remained elusive if under physiological conditions, monocytes contribute to the establishment of the post-natal and adult microglia population. Fate mapping studies using Runx1MerCreMer lineage tracing model, in which exclusively yolk sac-derived progenitors and their progeny are fluorescently labeled following a tamoxifen pulse at E7.25, have now established that microglia are derived from yolk-sac progenitors that generate a long-living population with self-renewal capacity (28). It was further demonstrated that microglia develop from erythro-myeloid progenitors (EMP) in a stepwise PU.1 and IRF8-dependent manner (29, 30) (Figure 1). The development of microglia and primitive yolk sac macrophages is completely dependent on colony-stimulating factor 1 receptor (Csf1r) signaling (28). Microglia are absent in Csf1r knock-out mice, while mice lacking functional Csf1 did not show the same severe phenotype (31, 32). This observation was later explained by the existence of a second ligand for Csf1r, namely IL34 (33) that is highly expressed in the brain (34). Microglia represent the only tissue-resident macrophages that are exclusively derived from yolk sac-derived progenitors. By contrast, tissue-resident macrophages in other organs such as Kupffer cells in the liver, alveolar macrophages in the lung, or Langerhans cells in the skin comprise mixed populations and are repopulated by cells originating from the fetal liver during definitive hematopoiesis (27, 35). In light of recent experimental insight, it became apparent that previous findings that indicated a contribution of blood-borne monocytes to the adult microglia pool were confounded by experimental caveats that conditioned the brain for engraftment of peripheral myeloid cells, such as irradiation or parabiosis bias (36). Mildner et al. demonstrated that the use of head shields during myoablative irradiation prior to bone marrow transplantation prevented the recruitment of bone marrow-derived cells into the brain (37). These findings were further supported by studies using parabiosis in mice without the need for irradiation (38). Although chimerism in the periphery reached about 50%, there was no evidence for recruitment of peripheral monocytes to the brain. Moreover, even in the context of inflammation, when monocytes contribute to the inflammatory milieu, blood-borne cells did not integrate into the long-term resident microglia pool (39). The microglia compartment seemed to recover from an internal pool instead. These findings are in line with previous observations demonstrating that peripheral macrophages do not transform and replace microglial cells in EAE models (20). In contrast to these findings, it was shown that under experimental conditions in which the microglial niche is completely vacant in response to microglia depletion strategies, bone marrow-derived cells enter the brain and differentiate into microglia (40, 41). Bruttger et al. recently took advantage of a Cx3cr1CreER-based system (Cx3cr1-iDTR mice) (42) that allows for conditional depletion of microglia without the necessity of generating bone marrow chimera (43). The authors demonstrated that the repopulating microglia arose exclusively from an internal CNS-resident pool. A contribution of bone marrow-derived cells was only observed in mice that were irradiated and additionally received a bone marrow transfer. Moreover, it was demonstrated that microglia self-renewal is dependent on IL1 signaling, while reconstitution from bone marrow precursor is IL1 independent. However, until recently the actual turnover rate of microglia in the brain remained elusive. Employing a multicolor fate-mapping model, the microfetti mouse [a microglia-restricted modification of the confetti mouse (44)], Tay et al. recently analyzed the rate of self-renewal of microglia in steady state, after induced CNS pathology and during the subsequent recovery phase. This study revealed heterogeneous rates of microglia replenishment in different brain regions (45). Following CNS damage, the authors found a shift from a pattern of random self-renewal within the microglial network toward a rapid expansion of selected microglia clones. This finding provides important insight into the question if microglia are recruited from adjacent regions to sites of CNS damage, or if clonal expansion results in microglial accumulation. Results obtained in the Microfetti mouse clearly favor the latter hypothesis. During the recovery phase in which microgliosis is resolved, the restoration of microglial cell density occurred through egress and apoptotic cell death (45).

Taken together, the field has reached consensus regarding the origin of microglia and the contribution of bone marrow precursors to the microglia pool under steady-state conditions. However, the debate on the functional contribution of yolk sac-derived microglia and blood-borne monocytes in CNS inflammation and their functional interplay is still in its infancy. As discussed in more detail in the following paragraphs, there is evidence that in response to inflammatory conditions associated with, e.g., irradiation, neurodegenerative disorders, or CNS cancer, the recruitment of monocytes or other bone marrow-derived progenitors can supplement the microglial pool. However, it remains unclear if the recruited cells persist and become an integral part of the microglial population, or if those cells represent a transient population that vanishes once the inflammatory stimulus is resolved. Another question that still needs to be addressed in more detail is, if yolk sac-derived microglia and bone marrow-derived macrophages (BMDM) exert redundant or cell type-specific functions in CNS pathologies and if the ontological origin determines responses against therapeutic intervention.

## Shaping of Cellular Identity by the Tissue Environment

**Figure 2:**
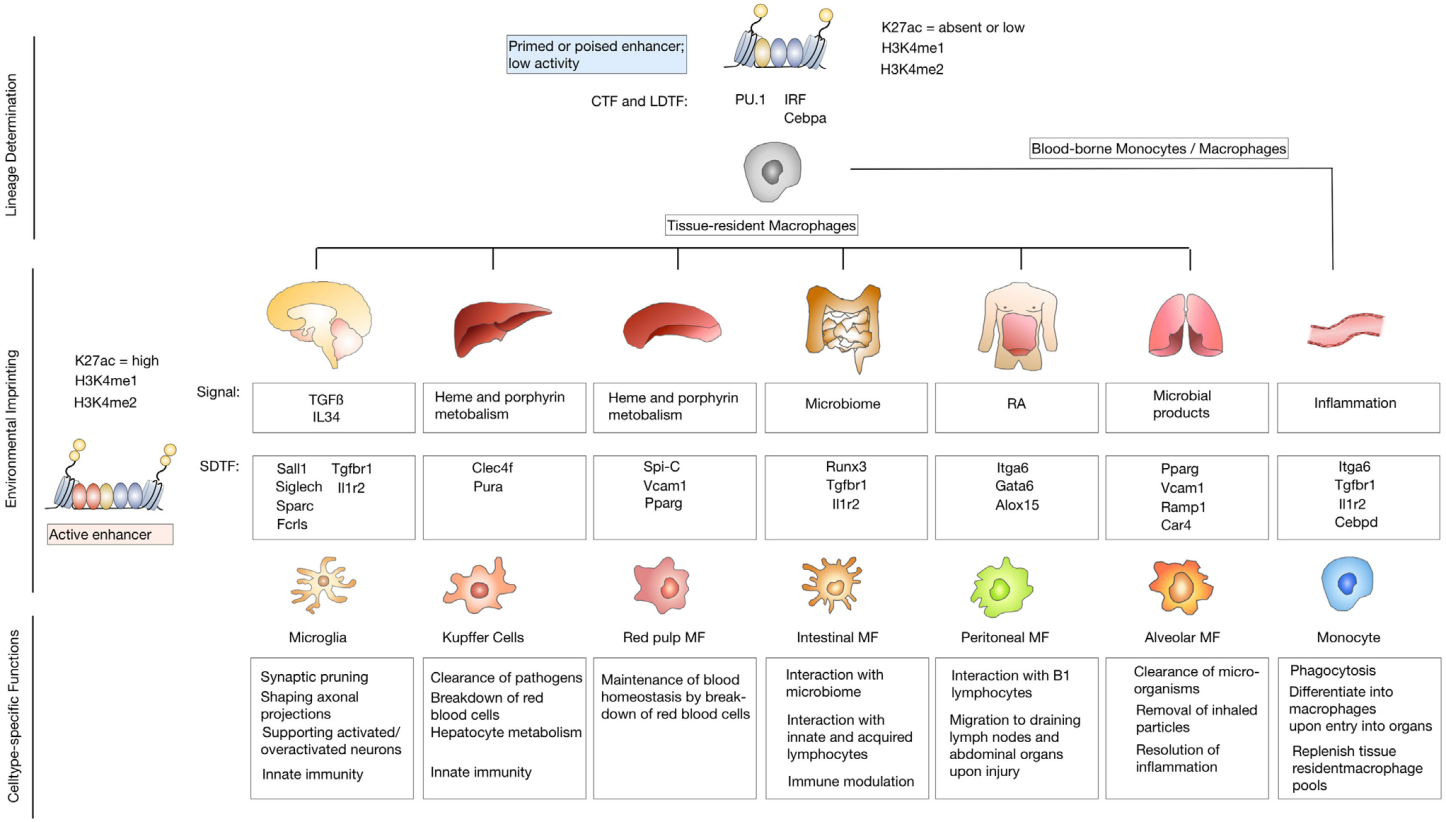
Environmental imprinting of tissue-resident macrophages. Differentiation of precursor cells into specific lineages is determined by binding of lineage-determining transcription factors (LDTFs) and collaborating transcription factors (CTFs) to closely spaced recognition patterns on the DNA. Binding of LDTFs and CTFs selects enhancers as primed or poised. Primed enhancers are marked with characteristic histone modifications such as histone lysine 4 monomethylation (H3K4me1) or dimethylation (H3K4me2). Poised enhancers are defined by the presence of histone H3 lysine 27 trimethylation (H3K27me3). Primed or enhancers show low activity due to the lack of enhancer RNA production or the presence of H3K27me3, that has to be removed to induce an active enhancer state (upper panel). Tissue-resident macrophage populations are exposed to unique environmental cues that lead to genetic and epigenetic imprinting based on signal-dependent transcription factors (SDTF) that bind and activate primed or poised enhancers. Active enhancers are marked by H3K4m1 or H3K4me2 and histone H3 lysine 27 acetylation (H3K27ac) (middle panel). Environmental imprinting induces cell-type-specific functions of different tissue-resident macrophage populations (lower panel).

To understand the imprinting of disease-associated states on microglia and monocyte-derived macrophage identity in more detail, it is important to first consider the effects of specialized tissue environments on tissue-resident macrophages. It is increasingly recognized that in addition to the ontological origin, environmental factors play a critical role in defining functionality of tissue-resident macrophages and determine the fate and persistence of cells in tissues. Consistent with their diverse locations and functions, tissue-resident macrophages in different organs display distinct gene expression profiles (46, 47). Several studies have already dissected the genetic and epigenetic imprinting of specific tissue-resident macrophages and identified a range of transcription factors that are essential for cell type restricted gene expression profiles, e.g., SpiC for red pulp macrophages (48, 49) and GATA6 for peritoneal macrophages (46, 50). Two recent studies undertook the effort to systematically characterize the genetic and epigenetic imprinting of tissue-resident macrophages in specific organ environments. Both studies used RNA sequencing in combination with chromatin immune-precipitation (Chip)-Seq (51) and assay for transposase-accessible chromatin (ATAC)-Seq (52) to identify enhancer regions that are coupled to gene expression and accessible chromatin (53, 54). The studies by Lavin and Gosselin indicate that tissue-resident macrophages share epigenetic structures and gene expression with other myeloid cell populations. Similarities within the lineage are largely determined by collaborating transcription factors (CTFs) such as PU.1 and lineage-determining transcription factors, including interferon regulatory factor family members and CCAAT/Enhancer-Binding-Protein (Cebp)-a (55–57). However, each tissue additionally has its unique gene expression profile that is controlled by changes in enhancer landscapes in response to environment-specific signals (Figure 2). Interestingly, both studies describe pronounced differences in enhancer landscapes among macrophage subtypes, while promoters are largely shared across different macrophage subpopulations and even between macrophages, monocytes, and neutrophils. It was demonstrated that microglia are most distinct from other tissue-resident macrophages in terms of their genetic landscape (53). This comparison also revealed that macrophage populations that are exposed to similar environmental cues converged to similar expression patterns. For example, Kupffer cells and splenic macrophages were shown to share a cluster of highly expressed genes that are enriched for gene ontology (GO) annotations, such as heme and porphyrin metabolism, indicating their role in erythrocyte turnover (48, 49). Similarly, small and large intestinal macrophages were shown to express genes enriched for GO annotations that reflect exposure to microbiota, such as response to bacteria and antigen processing. A more detailed comparison between microglia and peritoneal macrophages identified tissue-specific signals that determine the epigenetic and genetic imprinting of microglia and peritoneal macrophages. The genetic landscape of microglia is known to be strongly driven by the presence of TGFβ and IL34 (58, 59), while retinoic acid is a well-characterized environmental factor that dictates genetic imprinting of peritoneal macrophages and is essential for their development and function *via* GATA6 activation (60). The extent of tissue-specific cues on enhancer landscapes was further proven by transplantation experiments in which peritoneal macrophages were transferred to the lungs. Interestingly, the transferred tissue-resident macrophages lost most of their original tissue marks and acquired a tissue program based on their new host tissue (54).

In summary, identification of enhancer landscapes that are imprinted by specific tissue environments together with the notion that environmental cues can override ontological imprinting ultimately leads to the question, how blood-borne monocytes and macrophages are affected by the host tissue upon recruitment to sites of injury, inflammation, neurodegeneration, and neoplastic transformation and also, to which extent, the disease status dominates the imprinting of resident and recruited cell populations. The next paragraph will discuss recent findings from the field of neurodegenerative disorders, with a focus on Alzheimer’s disease (AD), and brain cancers that provide critical insight into the heterogeneity of disease-associated myeloid cells.

## Molecular Identities of Microglia and Macrophages in Brain Malignancy

The local tissue environment has been shown to sculpt macrophage transcriptional profiles and epigenetic states under steady state conditions. However, it remained unclear whether an inflammatory tissue environment may affect differently macrophage populations of distinct ontogenies. To answer this question, it is first essential to determine the extent of peripheral recruitment of myeloid cells to the CNS under distinct pathological conditions. A critical contribution of recruited myeloid cell populations has been proposed for a long time (61). However, given the number of experimental caveats including the requirement of radiation for bone marrow transplantation (36, 37) as well as the route for tumor cell implantation, which often relies on intracranial injection, it remained unclear if the observed infiltration is due to experimental manipulation or represents an integral part of disease progression. Another important aspect that has to be taken into account is species differences that impact the extent of infiltration of cells from the periphery. It was shown that rat models for ischemia or brain cancer show lower rates of infiltration of blood-borne cells than observed in mouse models (62, 63). There is also evidence that recruitment of blood-borne inflammatory cells in human brain cancers is less pronounced than in mouse models (64). New approaches that employ lineage tracing models or single-cell RNAseq provide an unbiased view on the extent of infiltration from the periphery and the disease-associated imprinting on different myeloid subpopulations. Moreover, a growing number of studies addresses these questions on patient-derived samples thereby excluding the possible effects of experimental artifacts and provide important insight into the clinical relevance of experimental data (65–68).

### Neurodegenerative Disorders

Neurodegenerative disorders share common features including neuronal loss that ultimately leads to cognitive decline and motor dysfunction, which is associated with the establishment of an inflammatory environment. The immune milieu is comprised predominately of brain-resident microglia, which is supplemented by infiltrating immune cells. While AD, Parkinson’s disease (PD), Amyotrophic Lateral Sclerosis (ALS), and multiple sclerosis (MS) are considered as neurodegenerative diseases, it is important to appreciate differences in the extent of peripheral involvement, a critical parameter to define neuroinflammation (69). ALS and MS are autoimmune inflammatory disorders of the CNS that lead to irreversible axonal damage and progressive neurological disability (70, 71). In case of ALS and MS, immune cell infiltration is causative. For example, in MS, acute demyelinating white matter lesions show myelin breakdown accompanied by infiltration of innate immune cells (i.e., monocytes) and adaptive immune cells (T- and B-lymphocytes) (72, 73). By contrast, symptoms of AD and PD should rather be regarded as innate immune reactions (74). Infiltration of lymphocytes only occurs at late disease stages when integrity of the BBB is lost. The critical contribution of CNS inflammation to disease progression in neurodegenerative disorders has been appreciated since the first description of the pathological parameters in particular due to the manifestation of microgliosis (15, 75). However, to date, it still remains controversial if CNS inflammation, in particular under conditions that trigger pronounced recruitment of myeloid cells from the periphery, is associated rather with disease amelioration or acceleration.

Alzheimer’s disease represents the most common form of dementia and AD pathology is characterized by extracellular deposition of amyloid-β peptides that leads to β-amyloid plaques, formation of neurofibrillary tangles composed of hyperphosphorylated tau protein, neuroinflammation, and neuronal loss (76). An accumulating number of studies seek to evaluate the functional role of brain-resident microglia and to dissect the contribution of recruited myeloid and lymphoid cells. While some studies propose a protective role of microglia in AD (77–79), other reports show that under disease conditions, microglia acquire pro-inflammatory properties that have been associated with disease acceleration (80–83). These conflicting data can at least in part be attributed to a high phenotypic and functional heterogeneity of the disease-associated myeloid cell population. For example, Mildner et al. reported distinct and non-redundant roles of microglia and other brain-associated myeloid cells in AD mouse models. This study revealed a dominant role of CCR2-expressing myeloid cells in β-amyloid clearance (84). Previous studies that aimed at dissecting the functional contribution of myeloid subpopulations in disease progression relied on analyses of bulk cell populations categorized by a limited number of markers and single time points for analyses often at end stage disease. Importantly, analysis of bulk populations limits the capacity in resolving the heterogeneity and complexity of the immune milieu within the CNS (85–87). To date, single-cell transcriptomics allows for an unbiased characterization of immune cell types and states, thus systematically resolving the complex heterogeneity of the disease-associated immune landscape in comparison to normal tissue or in response to therapy (30, 88–91). Two recent studies used single-cell RNA seq analysis to characterize the immune landscape at different stages of disease progression in AD using the 5XFAD model (92) or the CKp25 model (93). Using the CKp25 AD-like mouse model, Mathys et al. identified early- and late-response states that differ significantly from homeostatic microglia. The early-response microglia show enrichment of cell cycle genes and genes involved in DNA replication and repair indicating that microglia expansion occurs at early disease stages. At late stages of disease progression, immune-related pathways were dominant and an enrichment of interferon-related response genes was detected. Interestingly, the late stage clusters comprised heterogeneous populations. Based on gene expression signatures of different clusters identified at late stages, the authors conclude that those subpopulations could reflect exposure to type 1 or type 2 interferon, respectively (93). Using the 5XFAD model, Keren-Shaul et al. also identified two distinct microglia states [cluster II and III, referred to as disease-associated microglia (DAM)] in AD that were absent in normal brain. Compared to normal microglia, DAMs showed reduced expression of microglia core genes including the purinergic receptors P2ry12, P2ry13, Cx3cr1, and Tmem119 (58, 94). Concomitantly, DAMs showed enrichment of genes that are known as common risk factors for AD, including Apoe, Ctsd, Lpl, Tyrobp and Trem2. Gene set enrichment analysis indicated induction of lysosomal/phagocytic pathways, endocytosis and regulation of immune response. Interestingly, temporal resolution of the DAM phenotype manifestation indicated a two-step process. The first step appears to be accompanied by suppression of key regulators of microglial phenotype and function, such as Cx3cr1. The second stage was shown to be dependent on Trem2 and Tyrobp/Dap12. Analysis of the spatial localization of DAMs revealed close association to amyloid plaques. Given the enrichment of phagocytic and lipid metabolism pathways, the authors propose that DAM are involved in plaque clearance. The presence of DAM-like cells in AD patients has been demonstrated by histology as well as transcriptomic analysis (66, 92).

In addition to the investigation on DAMs in AD, Keren-Shaul et al. interrogated if DAMs are also present in other neurodegenerative pathologies, including a mouse model for ALS and aging. Interestingly, distinct myeloid subpopulations that showed similarities to DAMs in AD were observed also in response to aging and in ALS (92). Given the finding that progressive neurodegeneration leads to the induction of similar gene signatures in DAMs it is very interesting to compare the results by Keren-Shaul and Mathys. Both studies describe the occurrence of two distinct microglia subpopulations during disease progression that are distinct from the microglia state in the healthy brain. However, early and late disease-stage associated populations are not completely unrelated. Earlier stages rather represent a transient intermediate activation state as part of the reprogramming of homeostatic microglia in response to neurodegeneration. Interestingly, late-response microglia express increased levels of many genes that were also observed to be upregulated in DAM, suggesting a substantial similarity between the expression profiles of DAM and late-response microglia (92, 93). This observation is consistent with the idea that the DAM program may be a primed set of genes that is expressed in response to varied conditions of altered homeostasis. This is further supported by gene expression similarities between AD, ALS and aging. However, the identified populations also show important differences. For instance, Mathys et al. observed that many antiviral and interferon response genes were significantly upregulated in late-response microglia but not in DAM. Moreover, significant differences in the expression of both stage 1 and stage 2 DAM enriched genes were observed in late-response microglia. This was less pronounced in early-response microglia. Differences in gene signatures can be in part be explained by model-specific characteristics. However, this observation might also indicate that the distinct microglia states represent intermediate stages on a continuum of microglia reprogramming that ultimately converts protective/beneficial functions into neurotoxic functions (92, 93). A comparison of the observed signatures and phenotypes suggests that the early- and late-stage microglia represent the most naïve and most advanced population, respectively, while the DAM stage 1 and stage 2 might occur along the transition from early to late stage microglia. Mrdjen et al. took a similar approach as recently employed by Korin et al. for steady state conditions (11) to investigate the immune landscape in CNS inflammation using mass cytometry (95). The combination of CyTOF together with lineage tracing allowed the authors to identify different subsets of myeloid cells and the phenotypic changes in CNS immune cells during aging, AD and MS with definitive proof of the ontological origin. Microglia phenotypes observed by Mrdjen in an EAE model reflected an inflammatory phenotype that showed similarities to the phenotypes observed in aging and AD mouse models. This indicates a potential universal disease-associated microglial signature as recently proposed (96).

**Figure 3:**
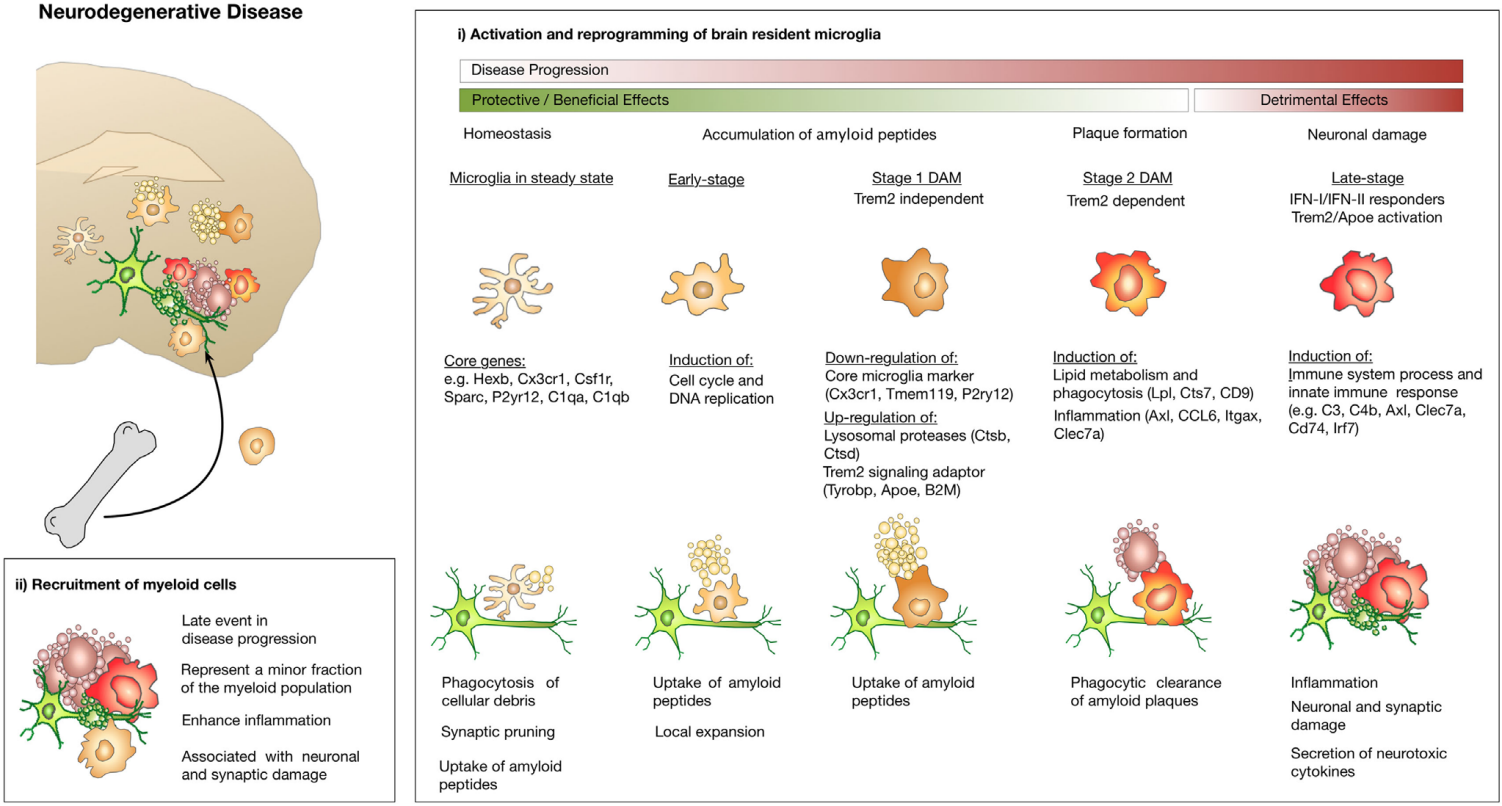
Microglia and macrophage activation states and effector functions at different stages of disease progression in neurodegenerative disease. (i) Microglia exert protective functions including phagocytosis of cellular debris, uptake of Aβ peptides, and clearance of amyloid plaques at early disease stages. Disease progression leads to changes in microglia functions that limit their ability to confine disease manifestation or even induces inflammatory activation states that cause neuronal and synaptic damage. Induction of Trem2/Apoe signaling was shown to mediate conversion of protective microglia into tissue damaging ones. Recruitment of macrophages from the periphery appears to occur at late disease stages and contributes to disease acceleration due to enhanced inflammation.

Although the studies by Keren-Shaul and Mathys both provide important insight into the molecular basis of DAMs or early- and late stage microglia, the beneficial or detrimental role of the respective subpopulations remains to be studied in more detail. Keren-Shaul et al. propose a protective function of DAMs due to their contribution in plaque clearance (92). Tyrobp and Trem2 are known to form a signaling complex associated with phagocytosis (97). Trem2 expression is known to be critical for clearance of neuronal debris and loss of function of Trem2 or Tyrbp (Dap12) are associated with dementias characteristic of neocortical degeneration observed in AD. In line with this interpretation, it has previously been reported that Trem2 is critical for microglia clustering and expansion around amyloid plaques and that the Trem2-mediated early microglial response limits diffusion and toxicity of amyloid plaques (79, 82). By contrast, Jay et al. demonstrated that Trem2 deficiency resulted in reduced infiltration of inflammatory myeloid cells and thereby ameliorated AD pathology at early stages (77) and exacerbated it at later stage (98). Hence, one possible explanation might be that DAM function has a transient beneficial impact during the initial phase of AD onset while later stages might be associated with rather detrimental effects. In light of recent insight on microglial stages in AD, Hansen et al. proposed a dichotomous role of microglia, with the detrimental microglia population occurring later in disease course at the time when synapse loss is observed and symptoms manifest (99). As depicted in Figure 3, microglia in steady state are protective and AD is prevented by constant scavenging of aβ peptides. Once the equilibrium is lost and Aβ levels accumulate, microglia phagocytose and clear Aβ aggregates. These protective activities involve activation of microglia to a DAM state in a Trem2-dependent manner. Genetic susceptibility or aging can lead to impaired microglia function. Accumulation of toxic amyloid plaques leads to tau pathology in stressed or damaged neurons, which induces an unconstructive and inflammatory state in microglia that causes deleterious neuronal damage (99) (Figure 3). This hypothesis is further supported by a recent study using the APP-PS1 model for AD, the ALS model SOD1G93A and an EAE model to investigate the role of the Trem2–Apoe complex in microglial dysfunction in neurodegeneration (96). In contrast to previous studies, Krasemann et al. proposed a neurodegenerative role of microglia in which the Trem2–Apoe pathway regulates the phenotypic switch from neuroprotective to neurodegenerative microglia. A negative feedback loop of Apoe to TGFβ suppressed homeostatic microglia concomitant with an induction of Bhlhe40 response genes including Clec7a, Lgals3, Gpnmb, Itgax, Spp1, Cl2, and Fabp5. Those recent data are in line with previous findings on the presence of functionally distinct or opposing microglia/monocytes populations in EAE models (83, 100). It was demonstrated that monocyte-derived macrophages initiate demyelination, while microglia rather clear debris. Gene expression analysis confirmed that macrophages are highly phagocytic and inflammatory, while microglia showed globally suppressed metabolism at early disease stages (83). Gao et al. proposed a dichotomy of the function for Tnfr2 in myeloid cells. It was shown that microglia-derived Tnfr2 signaling is associated with protective effects, while monocytic/macrophagic Tnfr2 stimulated immune activation and EAE initiation (100). Moreover, as discussed in the following paragraph on brain cancers, reprogramming of pro-inflammatory macrophages with anti-tumor functions into immune-suppressive, tumor promoting macrophages has been described for a comprehensive number of cancer entities which is often regarded as de-regulated wound healing programs.

### Primary and Secondary Brain Cancer

Massive infiltration of macrophages into tumors has been reported for a large proportion of primary tumor entities and metastasis. Macrophages represent the most abundant stromal cell type in many cancers and comprise up to 30–50% of the tumor mass including CNS cancers such as GBM (101) and brain metastases (102). Functional analyses indicate that the presence of tumor-associated macrophages (TAMs) fosters tumor growth, regulates metastasis and affects therapeutic response (103–105). Accumulation of TAMs is often associated with poor patient prognosis (106, 107).

To date only few studies have looked at the origin and fate of macrophages during cancer progression at the primary site and in metastasis. One of the first reports that systematically dissected the cellular and molecular origin of tumor-associated macrophages employed parabiosis experiments in the PyMT mouse model for breast cancer (108). The authors demonstrated that tumor development triggers a unique innate immune response that is characterized by the differentiation of inflammatory monocytes into tumor-associated macrophages (TAMs). Terminal differentiation of monocytes into TAMs occurred in a notch-dependent manner *via* the recombination signal binding protein for immunoglobulin regulator (Rbpj) (109). Monocyte-derived TAMs showed pronounced transcriptional differences compared to resident mammary-tissue-macrophages (MTM). TAM expansion during breast cancer progression led to a loss of MTMs (108). By contrast, it was recently demonstrated in PDAC models for pancreatic cancer that tissue-resident macrophages persist and undergo significant expansion. TAMs in PDAC tissues adopted a transcriptional program that is associated with cell proliferation (110). This effect was shown to be enhanced by tissue-resident macrophages derived from the yolk sac or fetal liver, but not by HSC-derived monocytes/macrophages. Consequently, macrophages of different ontological origins had different impact on tumor progression in the PDAC model. Loss of monocyte-derived macrophages only showed marginal effects on tumor progression, while depletion of tissue-resident macrophages significantly reduced tumor progression (110). The ontological origin of TAMs in primary brain cancer was investigated in several recent studies using different glioblastoma (GBM) mouse models (101, 111–113). The study by Muller et al. used bone marrow transplantation strategies with head protected irradiation (HPI) in the GL261 model in direct comparison to total body irradiation (TBI) (113). Avoiding previously reported irradiation bias in the head region, the authors demonstrate that recruited macrophages contribute only at later stages to the tumor mass and constitute around 25% of the myeloid population. Interestingly, tumor progression in TBI-treated mice was accelerated compared to the HPI cohort, suggesting that recruited macrophages contribute in promoting tumor growth, yet their infiltration might be predominately caused by impact of IR (113). In order to fully circumvent the necessity of IR, two recent studies employed a genetically engineered model for proneural GBM and the GL261 model in combination with different lineage tracing models to discriminate ontologically distinct subpopulations (111, 112). Using the Cx3cr1^GFP/wt^:Ccr2^RFP/wt^ double knock-in model (114, 115) (see also Table 1), Chen et al. demonstrated that Cx3cr1^lo^Ccr2^hi^ monocytes are recruited to GBM, where they differentiate into Cx3cr1^hi^Ccr2^lo^ macrophages and Cx3cr1^hi^Ccr2^neg^ microglia-like cells. In contrast to the results by Muller et al., recruitment of bone marrow-derived monocytes/macrophages was reported to occur at early stages of GBM initiation. Recruited macrophages were predominantly localized to perivascular areas, while microglia were found in peri-tumoral regions. Quantification of the extent of infiltration suggested that recruited macrophages constitute up to 85% of the TAM population, with the remaining 15% being represented by microglia. A possible explanation for the discrepancy of the observed influx in both studies might be differences in tissue harvest strategies. Chen et al. focused their analysis on macro-dissected tumor areas while Muller et al. processed the entire tumor-bearing hemisphere with considerable involvement of adjacent non-tumor-bearing brain parenchyma. Moreover, it is important to note that the study by Muller et al. provided evidence that GBM-associated microglia upregulate CD45 and represent an inherent part of the CD45^hi^ population in the tumor context. Upregulation of CD45 expression on microglia was previously demonstrated in response to different inflammatory stimuli (116) and underlines the fact that CD45 levels as proposed by Ford et al. (117) can only be used to discriminate macrophages and microglia under steady-state conditions while it is not suitable under inflammatory conditions. It was also demonstrated that activated microglia including GBM-associated microglia downregulate Cx3cr1 (112). The gating strategy employed by Chen et al. relied on the discrimination of macrophages and microglia by CD45 expression levels and used a mouse model that is based on Cx3cr1 and Ccr2 promoter activity for reporter gene expression (111). Hence, it is possible that the CD45^hi^Cx3cr1^med^ population represents a mixed population of microglia that upregulate CD45 concomitant with Cx3cr1 downregulation.

**Table 1:** Lineage tracing models and marker to distinguish microglia and monocyte-derived macrophages in the brain.

Approach	Cell type specificity	Principle	Advantages	Limitations	Reference
**(a) Transplantation models**					

BMT; TBI	BMDM	HSC source of blood monocytes is replaced with modified/labeled HSCs	High chimerism No time consuming crossing into genetic disease models	Variability in myeloablation and reconstitution Artificial engraftment of BM cells in the CNS	(113)

BMT; HPI	BMDM	HSC source of blood monocytes is replaced with modified/labeled HSCs	High chimerism No time consuming crossing into genetic disease models	Variability in myelo-ablation and reconstitution	(37, 113)

BMT; Busulfan	BMDM	HSC source of blood monocytes is replaced with modified/labeled HSCs	High chimerism No time consuming crossing into genetic disease modelsChemical myeloablation No irradiation	Variability in myeloablation and reconstitution	(112)

Parabiosis	BMDM	HSC source of blood monocytes is replaced with modified/labeled HSCs	Constant influx of donor cells No myeloablationrequired No time consuming crossing into genetic disease models	Technically challengingLow chimerism	(39)

**(b) Genetic lineage tracing models**					

Ccr2^RFP/wt^; Cx3cr1^GFP/wt^	Monocytes (red) MG (green)	Differential labeling of Cx3cr1^hi^: Ccr2^neg^ MG (green) and of Cx3cr1^lo^:Ccr2^hi^ monocytes (red)	MG and monocytes contain reporter for labeling	Recruitment to the brain leads to increased Cx3cr1 levels in monocyte-derived macrophages Ccr2 expression is downregulated in monocyte-derived macrophage upon differentiation	(114, 115)

Flt3-Cre	Monocytes and HSC-derived monocyte precursors	Label/modification induced in Flt3^+^ monocyte precursors	Useful for lineage tracing of myeloid precursors Useful complementary approach to MG restricted lineage tracing	Cre expression or transmittance restricted to male mice	(118)

Cx3cr1-Cre^ER^	MG	Recombination is induced in all Cx3cr1^+^ cells upon tamoxifen pulse. Long-living MG retain the label/modification. While monocytes vanish and are replenished from precursors that were generated after Cre recombination in response to the tamoxifen pulse	Long-term labeling/modification is restricted to MG	Spontaneous modification reported in one model Low recombination in mMF (40–50%)	(42)

Sall1-Cre^ER^	MG	Label/modification induced in Sall1^+^ MG	Sall1 expression is stable also in response to different stimuli	Targeting of non-hematopoietic cells in the liver, heart and kidney	(119)

**(c) Cell-type restricted marker expression**					

CD45	MG^lo^ BMDM^hi^	MG display lower surface expression	No requirement for combination of several markers	Activated in MG upregulate CD45 BMDM in brain malignancies downregulate CD45	(112, 113, 120)

Tmem119P2ry12Siglech	MG^hi^ BMDM^lo^	MG show high expression	Applicable for mouse and human	Downregulation in MG during activation BMDM in GBM show increased expression	(58, 94, 112)

Sall1	MG^hi^ BMDM^lo^	MG show high expression	Applicable for mouse and human Stable expression level at different activation levels	Low expression found on non-leukocytes in the liver, heart, and kidney	(119)

Itga4/Cd49d	MG^lo^ BMDM^hi^	BMDM show high expression	Applicable for mouse and human Stable expression level at different activation levels	Expression found on T cells	(112)

*BMT, bone marrow transplantation; TBI, total body irradiation; HPI, head protected irradiation; BMDM, bone marrow-derived macrophage; HSC, hematopoietic stem cell; MG, microglia; BM, bone marrow; CNS, central nervous system*.

**Figure 4:**
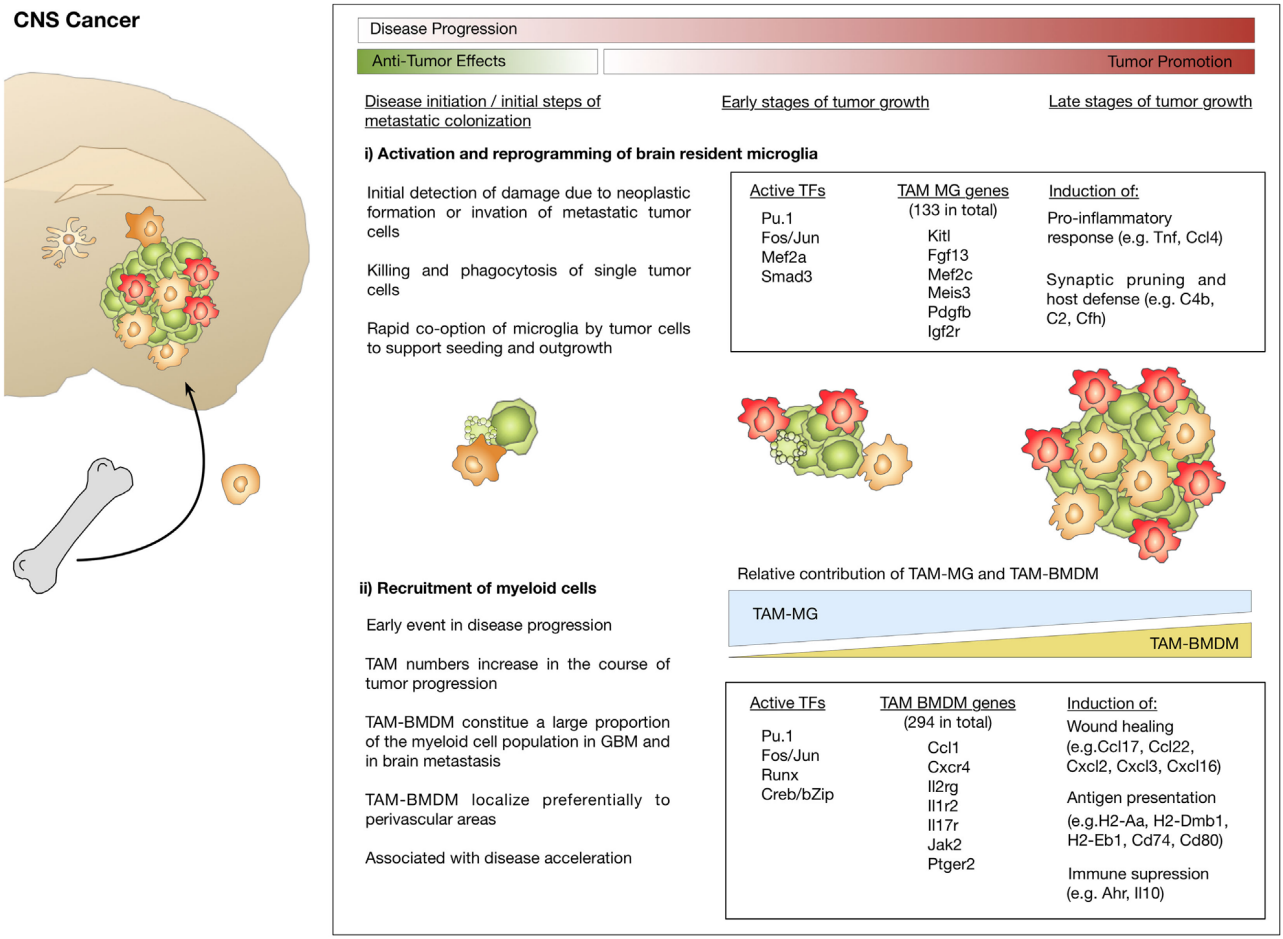
Microglia and macrophage activation states and effector functions at different stages of disease progression in brain cancer. (i) Initial stages during neoplastic transformation in primary brain cancers or tumor cell seeding in brain metastasis are detected by microglia. As part of their role in host defense, microglia induce apoptosis in cancer cells. Tumor cells that escape microglia-mediated killing rapidly co-opt them and exploit their function to foster tumor growth. (ii) Brain tumor formation is associated with pronounced recruitment of macrophages from the periphery that starts at early stages and leads to disease acceleration. Transcriptomic analysis identified gene signatures that define tumor-associated microglia (TAM-MG) and macrophages (TAM-BMDM). The respective signatures indicate that TAM-MG rather induce pro-inflammatory responses and exert host defense functions while TAM-BMDMs were associated with wound healing, antigen presentation, and immune suppression.

Bowman et al. used a comprehensive set of different lineage tracing models to unravel the extent of macrophage recruitment to GBM and brain metastasis (112). The lineage tracing models used in this study were based on specific labeling of microglia or BMDM using the tamoxifen inducible Cx3cr1CreER-IRIS-YFP;R26-LSL-TdTom model (42) or the Flt3Cre;R26mTmG model (118) (see also Table 1). Using these complementary lineage-tracing strategies, it was demonstrated that BMDMs contribute to the TAM pool in different GBM and brain metastasis models in the absence of IR. BMDMs constituted approximately 50% of the TAM population in GBM and 25% in brain metastasis. RNA sequencing of FACS sorted myeloid populations revealed distinct clustering of all TAM populations from normal microglia and monocytes. Within the TAM cluster, further cell and tumor type-specific clusters were identified (112). The TAM cluster showed enrichment of cell cycle related genes, upregulation of complement-related factors, extracellular matrix components, proteases, lipid metabolism mediators, and clotting factors. Interestingly, the authors found several microglia-enriched genes (e.g., Tmem119, Olfml3, Lag3, Jam2, and Sparc) to be upregulated in TAM-BMDM, while other microglia genes (e.g., Sall1, P2yr12, and Mef2c) were not induced in TAM-BMDM (112). These results further indicate that macrophages acquire tissue-resident gene expression upon infiltration into foreign tissue as previously proposed by Lavin et al. (54) and Gosselin et al. (53). Analysis of the epigenetic imprinting of TAM-MG and TAM-BMDM revealed enrichment of Fos/Jun and Pu.1 binding sites in both populations. In addition to those shared motifs, it was demonstrated that TAM-BMDM showed enriched enhancer usage for Runx and Creb/bZip motifs, while TAM-MG peaks were enriched for Smad3 and Mef2a. Interestingly, based on enriched genes it appears that TAM-MG rather exert pro-inflammatory functions as evident by an upregulation of cytokines such as Tnf and Ccl4 as well as classical complement components (e.g., C4b, C2, and Cfh), a pathway that was previously shown to be associated with synaptic pruning and host defense (121). By contrast, TAM-BMDM showed gene signatures that indicate functions in wound healing, antigen presentation and immune suppression (112) (Figure 4). Insight into the genetic and epigenetic landscape of TAM-MG and TAM-BMDM in GBM provides evidence for complex networks of tissue and disease imprinting that at least in part can be attributed to the ontological origin of the cells and can be linked to functional differences. In the future, it will be very interesting to analyze in more detail the heterogeneity of different subpopulations at different stages of disease progression as recently done for AD mouse models and to perform functional validation of the proposed mechanisms for each subpopulation. Moreover, it is still unknown if brain tumors of different origin such as oligodendroglioma or brain metastasis induce similar genetic and epigenetic imprinting like gliomas or if the signatures are fundamentally different.

In addition to the important insight into the identity of TAM populations in GBM, Bowman et al. took advantage of the RNAseq data obtained from lineage tracing models to identify markers that discriminate macrophages and microglia in a tissue- and disease independent manner. The authors identified and validated Itga4/CD49d that is specifically repressed in microglia, as a marker to distinguish BMDMs from microglia in primary and metastatic brain cancer in mouse models as well as human samples (112). A list of lineage tracing models and markers that allow for discrimination of microglia and recruited myeloid cells and highlights advantages and limitations is provided in Table 1. For review see also (122).

Within the last few years, a growing number of studies performed in-depth analyses on GBM patient samples. While the primary focus of these studies was rather on tumor cell centric questions, the results obtained from GBM sequencing allow conclusions on the tumor microenvironment. Wang et al. reported increased infiltration of macrophages during disease progression with the highest extent of macrophage/microglia accumulation in mesenchymal GBM (MES) versus non-MES (68). Moreover, NF1 loss that is frequently found in MES correlated with increased infiltration of macrophages/microglia. Venteicher et al. performed single-cell RNAseq experiments on GBMs with different IDH mutational status. In line with results from mouse models of GBM, it was reported that also in human GBM, the balance between microglia to macrophages shifts toward a higher representation of macrophage programs over microglia signatures (65). This effect was noted by downregulation of microglia core genes, such as CX3CR1, P2RY12, P2RY13, and SELPLG, concomitant with upregulation of macrophage-like signatures including increased expression of CD163, IFITM2, IFITM3, TAGLN2, F13A1, and TGFB1. Microglia and macrophages displayed signatures that reflected an inflammatory program consisting of cytokines (IL1, IL8, TNF), chemokines (CCL3, CCL4), NfkB-related genes as well as immediate early genes. In line with previous reports, expression analysis suggests that the GBM environment alters expression profiles of macrophages, thus reducing their transcriptional difference from microglia. Interestingly, the authors also identified a range of factors that correlated with increased macrophage infiltration. Of those 24 identified genes, three were components of the complement system (C1A, C1S, C4A) (65). Similarly, Darmanis et al. correlated gene expression within in the immune cell cluster (containing >95% macrophages/microglia and approximately 4.5% dendritic cells) with macrophage and microglia core genes to classify the cells into macrophages and microglia (67). Consistent with data obtained in GBM mouse models, it was shown that cells with macrophage-like signatures were found rather within tumor lesions, while microglia were localized at the tumor edge (67, 111). Interestingly, as previously proposed by Bowman et al., analysis of gene signatures revealed that more pro-inflammatory markers (e.g., CCL2, CCL4, TNF, IL6R, and IL1A/B) were expressed in the tumor periphery, whereas anti-inflammatory marker (e.g., IL1RN and TGFBI) were enriched in the tumor core (67, 112).

Taken together, while controversy remains on the extent of peripheral recruitment to GBM at different stages of tumor progression, there is accumulating evidence that the ontological origin of myeloid cells in brain cancers impacts the nature of genetic programs that are induced. Tumor-specific education of myeloid cells is expected to determine their effector functions during disease progression.

### Lessons Learnt From Neurodegenerative Disorders and Brain Cancers

A series of recent studies utilized in depth transcriptomic analysis to molecularly define myeloid subpopulations at different stages of disease progression. However, a systematic analysis with the aim to interrelate the findings from different datasets has been missing. To close this gap of knowledge, Friedman et al. performed a comprehensive meta-analysis of purified mouse CNS myeloid cell profiles from different conditions across multiple studies including ischemic, infectious, inflammatory, neoplastic, demyelinating, and neurodegenerative conditions (123). Importantly, it was noted that in all comparisons microglia/brain-associated myeloid cell enriched genes that distinguish them from myeloid cells/macrophages from the periphery were downregulated. It appears that in response to any perturbation, including normal aging, the genes that separate microglia from other macrophages and are, thus, likely involved in microglia function, show reduced expression. Friedman et al. identified several modules that are shared across pathological conditions, including the neurodegeneration-associated module with high similarities to DAM (92, 123). In addition to the DAM-like population, a unique microglia subset that expressed an interferon-related gene module was identified. This module showed increased representation with progressive β-amyloid pathology. This is in line with the distinct clusters observed in the CKp25 model that showed enrichment of interferon-responding genes and likely reflect subpopulations depending on exposure to type 1 or type 2 interferons (93, 123). Moreover, the authors compared data sets from mouse models with human samples to evaluate the translational capacity of the experimental data. Compared to mouse models, a greater range of cell-type variability was observed in the comparison of healthy and diseased human tissues, indicating that CNS inflammation in human disease is more pronounced than in commonly used mouse models (123).

Taken together, data obtained from studies on neurodegenerative disorders and brain cancers suggest that there is considerable overlap in certain tissue-specific gene signatures that are induced within the brain environment in response to disturbance of tissue homeostasis. This is apparent in the phenomenon that gene signatures in microglia and macrophages become more similar once both cell types are present in the brain (123). Moreover, a number of genes or pathways were found to be similarly dysregulated in neurodegenerative disorders and brain cancers and there is evidence of dichotomous roles of myeloid subpopulations/activation states under multiple pathological conditions. At early stages, myeloid cells are considered as protective cells that preserve tissue integrity by scavenging debris or eliminating intruders as part of their host defense mechanism. Advanced disease stages are rather associated with detrimental effects that cause tissue damage, neuronal loss and promote tumor growth (Figures 3 and 4) (99, 124). Neoplastic formation leads to a more rapid switch from beneficial to damaging effects and the extent of recruitment from the periphery appears to be more pronounced in brain cancers compared to neurodegeneration. It is increasingly recognized that tumor cells rapidly co-opt stromal cells and functionally reprogram their environment to generate a cancer permissive niche to foster tumor growth (124, 125). In this process, tumor cells are known to exploit housekeeping functions of stromal cells, such as host defense or wound healing mechanisms for their own benefit (126, 127). By contrast, recruitment of peripheral myeloid cells in neurodegenerative diseases such as AD appears to occur at later stages of disease progression and to a lesser extent. Protective programs of microglia have been shown to be preserved for an extended period during disease progression and to contribute in limiting disease propagation. However, at late disease stages, microglia function is no longer sufficient to prevent detrimental pathological events (99). Moreover, there is increasing evidence that dysregulation of certain pathways, e.g., the Trem2–Apoe pathway, represent a switch from protective to damaging effects (96).

As discussed in more detail in the next paragraph, detailed mechanistic insight is critical to develop therapeutic strategies that are targeted against aberrant functions of individual subpopulations at defined stages of disease progression as otherwise physiologically essential functions or protective programs might be blocked which consequently results in minimal therapeutic efficacy and adverse effects.

## Perspectives for Therapies Targeting Microglia/Macrophages in Neurodegenerative Disease and Brain Cancer

Macrophages/microglia-targeted therapies are emerging in the field of neurodegenerative disorders and cancer (128, 129). The rationale for environment-targeted therapies is based on high abundance of stromal cells in different pathologies as well as their critical impact on disease outcome. Moreover, it was reasoned that the risk for acquired resistance is lower in genetically stable non-malignant cells, compared to genetically instable cancer cells. Recent analysis of the phenotypic and functional heterogeneity of macrophage/microglia subpopulations in neurodegeneration and cancer clearly indicate that macrophage/microglia-targeted therapies have to be based on their disease-associated specificities to achieve high therapeutic efficacy without inducing adverse effects. A variety of macrophage/microglia-targeted strategies have been tested in pre-clinical settings using genetic and pharmacological approaches. Several inhibitors have already entered phase 2 clinical trials (130). Most of those strategies aim at either blocking the recruitment of macrophages or depleting them (131). Given the importance of CSF1R downstream signaling for the differentiation and survival of macrophages and microglia, many studies tested the efficacy of blocking the ligands (CSF1 and IL34) or the receptor. However, most of these studies reported no or low efficacy when used as monotherapy, while combination of CSF1R inhibition with standard of care (e.g., chemotherapy or irradiation) led to synergistic anti-tumor effects in, e.g., glioma and breast cancer (132, 133). By contrast, Pyonteck et al. demonstrated that monotherapy with the CSF1R inhibitor BLZ945 resulted in improved survival and tumor regression in a model for proneural GBM. CSF1R inhibition in this model did not result in depletion of TAMs. Instead, TAMs showed reduced expression of several M2-like markers. The authors concluded that CSF1R-induced depolarization of TAMs might be more efficient than depletion of TAMs (101, 131). The same group recently demonstrated that long-term CSF1R inhibitor treatment led to acquired resistance driven by a compensatory IGF1–IGF1R signaling loop between macrophages and tumor cells, resulting in enhanced glioma cell survival and invasion (134). CSF1R inhibitors are currently in clinical trials to test their efficacy in GBM patients. The clinical trial using the CSF1R inhibitor PLX3397 in recurrent GBM (NCT01349036) was recently completed. PLX3397 was well tolerated but showed no efficacy in the recruited patient cohort (130). Additional studies that test CSF1R inhibitors in combination with standard of care or immune therapy are currently ongoing [e.g., BLZ945 with PRD001 anti-programed cell death-1 (PD1) in solid tumors including recurrent GBM (NCT02829723) and PLX3397 with temozolomide and radiotherapy in newly diagnosed GBM (NCT01790503)].

Colony stimulating factor 1 receptor inhibition was also tested in models for neurodegenerative disease to limit damaging neuroinflammation at disease end stage. Elmore et al. depleted virtually the entire microglia pool using the CSF1R/c-KIT inhibitor PLX3397 with no impairment of behavior and cognition. After withdrawal of the inhibitor, microglia rapidly repopulated the brain, returning to normal numbers within two weeks. Replenishment of microglia after CSF1R inhibition occurred from nestin^+^ progenitor cells that induced expression of microglia-associated genes such as Iba1, Cx3cr1, Tmem119, Siglech, Pu.1, and Trem2 (135). Repopulating microglia were shown to be functional and responsive to inflammatory challenge similar to resident microglia (136). Hence, CSF1R-mediated microglia depletion might provide a powerful tool to resolve tissue destructive inflammation. Using the selective CSF1R inhibitor PLX5562, it was demonstrated that treatment with lower doses (leading to 30% depletion) strongly reduced microglia accumulation at amyloid plaques in the 3xTg-AD model. While plaque burden was not reduced, treatment led to improved cognition (137). Interestingly, CSF1 signaling can be regulated by Trem2, which suggests that effects of Trem2 on microglia could in part be mediated by the CSF1 signaling cascade (82). Likewise, Trem2 deficiency resulted in reduced accumulation of microglia around amyloid plaques. Krasemann et al. demonstrated that the Trem2–Apoe pathway induces a switch from a homeostatic to neurodegenerative phenotype in microglia. It was, therefore, proposed that modulation of the neurodegenerative phenotype through targeting of the Trem2–Apoe pathway might allow restoring homeostatic microglia and treat neurodegenerative disorders (96).

The recent advances in our understanding of niche-, stage-, and activation state-dependent roles of microglia and macrophages in different brain malignancies certainly open new opportunities for therapeutic intervention. Stage- or population-specific gene signature might also serve as new prognostic biomarkers to identify high-risk patients based on inflammatory fingerprints. Thorough functional validation of candidate genes that are associated with dysregulated microglia/macrophage function is needed to identify druggable targets for therapies aiming at reverting disease-promoting into protective effects or to maintain beneficial house-keeping functions as proposed for the Trem2–Apoe pathway.

## Concluding Remarks

The history of microglia in brain malignancies started almost a century ago with their initial description by Pio del Rio-Hortega. Their ontological origin and biological function in health and disease has been controversially discussed ever since. While in-depth analysis down to the single-cell level provided critical insight into the heterogeneity of microglia and their blood-borne counterparts, we are just at the beginning to understand how different subpopulations or activation states regulate CNS homeostasis at steady state and how aberrant functions affect disease progression. Further investigation of the mechanisms that drive microglia and macrophage dysregulation will hopefully provide scientific rationale for the development of novel targeted therapies that provide better treatment options for patients to improve prognosis and quality of life.

## Author Contributions

The author confirms being the sole contributor of this work and approved it for publication.

## Conflict of Interest Statement

The author declares that the research was conducted in the absence of any commercial or financial relationship that could be construed as a potential conflict of interest.
